# Clinical feature of severe fever with thrombocytopenia syndrome (SFTS)-associated encephalitis/encephalopathy: a retrospective study

**DOI:** 10.1186/s12879-021-06627-1

**Published:** 2021-09-03

**Authors:** Ying Xu, Mingran Shao, Ning Liu, Danjiang Dong, Jian Tang, Qin Gu

**Affiliations:** 1 Department of Intensive Care Unit, The Affiliated Nanjing Drum Tower Hospital of Nanjing University Medical School, 210008 Nanjing, China; 2 Department of Radiology, The Affiliated Nanjing Drum Tower Hospital of Nanjing University Medical School, 210008 Nanjing, China

**Keywords:** Encephalitis, Encephalopathy, Severe fever with thrombocytopenia syndrome, Cerebrospinal fluid, Bacteremia, Vasopressor, Amylase

## Abstract

**Background/objective:**

Severe fever with thrombocytopenia syndrome (SFTS) cause encephalitis/encephalopathy, but few reports were available. We aimed to investigate the incidence of encephalitis/encephalopathy in SFTS patients and to summarize clinical characteristics, laboratory findings and imaging features.

**Methods:**

We conducted a retrospective review of all patients with confirmed SFTS admitted to Nanjing Drum Tower Hospital, a tertiary hospital in Nanjing City, China, between January 2016 and July 2020. The patients were divided into two groups according to whether they had encephalitis/encephalopathy: encephalitis/encephalopathy group and non- encephalitis/encephalopathy group. Clinical data, laboratory findings, imaging characteristics, treatments and outcomes of these patients were collected and analyzed.

**Results:**

A total of 109 SFTS patients with were included, of whom 30 (27.5 %) developed encephalitis/encephalopathy. In-hospital mortality (43.3 %) was higher in encephalitis/encephalopathy group than non-encephalitis/encephalopathy group (12.7 %). Univariate logistic regression showed that cough, wheezing, dyspnoea, respiratory failure, vasopressors use, bacteremia, invasive pulmonary aspergillosis (IPA) diagnoses, PCT > 0.5 ug/L, CRP > 8 mg/L, AST > 200 U/L and serum amylase level > 80 U/L were the risk factors for the development of encephalitis/encephalopathy for SFTS patients. Multivariate logistic regression analysis identified bacteremia, PCT > 0.5 mg/L and serum amylase level > 80 U/L as independent predictors of encephalitis/ encephalopathy development for SFTS patients.

**Conclusions:**

SFTS-associated encephalitis/encephalopathy has high morbidity and mortality. it was necessary to strengthen the screening of CSF testing and brain imaging after admission for SFTS patients who had symptoms of encephalitis/encephalopathy. SFTS patients with bacteremia, PCT > 0.5 ug/L or serum amylase level > 80 U/L should be warned to progress to encephalopathy.

## Background

Severe fever with Thrombocytopenia syndrome virus (SFTSV), a novel Banyangvirus genus in the Phenuiviridae family, was first discovered in China in 2009 [1]. Since then, the virus has also been isolated in South Korea, Japan, Vietnam, Taiwan, Myanmar, Thailand and Pakistan [2–7]. SFTS is a kind of viral hemorrhagic fever with high fatality rate and patients with SFTS show common symptoms such as a sudden fever, vomiting, stomachache, diarrhea, muscle soreness and some hemorrhagic symptoms [8, 9]. Severe cases can cause central nervous system (CNS) symptoms and are thought to be related to the severity of the disease[8] . The fatality rate of SFTS patients with encephalitis was reported as high as 44.7 % [10]. However, to date, few reports of CNS complications in SFTS patients were few and have a detailed CSF testing [10, 11]. Therefore, we investigated the clinical and laboratory findings in SFTS patients with confirmed encephalitis/encephalopathy.

## Methods

### Study population

We conducted a retrospective review of the patients with confirmed SFTS admitted to Nanjing Drum Tower Hospital, a tertiary hospital in Nanjing, China, between January 2016 and July 2020. The requirement for informed consent by individual patients was waived by Ethical Committee of Drum Tower Hospital affiliated with the Medical School of Nanjing University given the retrospective nature of the study.

### Diagnostic criteria for SFTS and SFTS-associated encephalopathy/ encephalitis

The criteria for confirmed SFTS were defined by (a) acute fever, (b) thrombocytopaenia, (c) detection of SFTSV RNA using polymerase chain reaction, detection of IgM against SFTSV, or isolation and culture positive of SFTSV [12]. The criteria for clinical diagnosed encephalopathy/encephalitis was defined as meeting the following criteria: (1) major criterion (required): Patients presenting to medical attention with altered mental status (defined as decreased or altered level of consciousness, lethargy or personality change) lasting ≥ 24 h with no alternative cause identified. (2) minor criteria (2 required for possible encephalitis; ≥ 3 required for probable or confirmed encephalitis): ① documented fever ≥ 38° C (100.4 °F) within the 72 h before or after presentation; ② generalized or partial seizures not fully attributable to a preexisting seizure disorder; ③ new onset of focal neurologic findings; ④ CSF WBC count ≥ 5/cubic mm; ⑤ abnormality of brain parenchyma on neuroimaging suggestive of encephalitis that is either new from prior studies or appears acute in onset; ⑥ abnormality on electroencephalography that is consistent with encephalitis and not attributable to another cause [13]. The criteria for clinical diagnosed SFTS-associated encephalopathy/encephalitis: meet SFTS criteria and encephalopathy/encephalitis simultaneously.

### Clinical data collection

Investigators collected clinical data through the electronic medical record system in Nanjing Drum Tower Hospital, a tertiary hospital in Nanjing, China, including demographic data, underling diseases, clinical symptoms and laboratory findings, comorbidity, treatment and overall prognosis were also recorded.

### Statistical analysis

Data were analyzed with SPSS 21.0 (SPSS Inc., Chicago, USA) for all statistical analysis. Data are reported as percentage for categorical variables and as mean ± standard deviation (SD) or median with interquartile range (IQR), as appropriate, for continuous variables. Chi-square test or Fisher’s exact test were used for categorical variables. T-test was used for continuous variables, as propriate. All tests of significance were 2-sided, and p < 0.05 was considered statistically significant. Univariate and multivariate logistic regression analyses were undertaken to examine risk factors for the development of encephalitis/encephalopathy. The results are reported as adjusted odds ratio of death with corresponding 95 % confidence intervals.

## Results

### Clinical characteristics

**Fig. 1:**
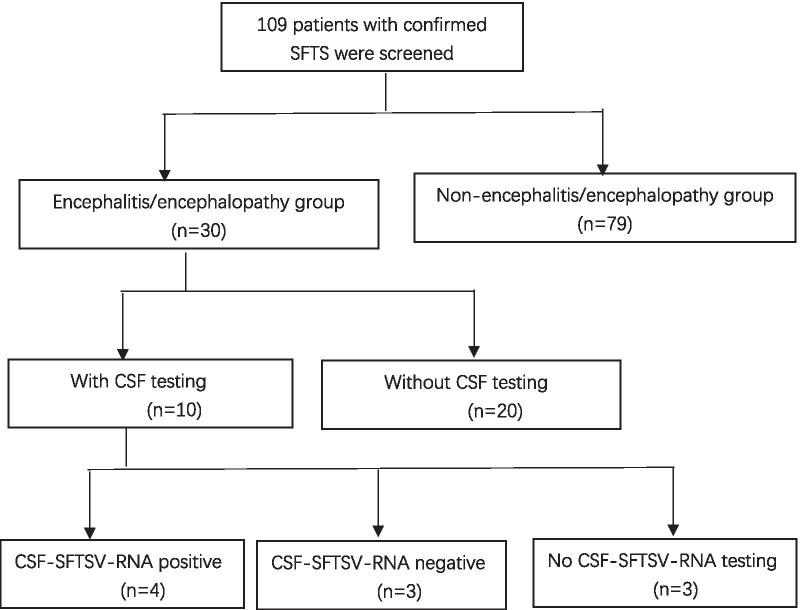
Schematic flow chart of patient enrolment. *SFTS* severe fever with thrombocytopenia syndrome, *CSF* cerebrospinal fluid

Between January 2016 and July 2020, a total 109 cases were confirmed with SFTS diagnosed by positive detection of SFTSV through polymerase chain reaction (PCR) for RNA. Of these 109 patients, 30 (27.5 %) cases were diagnosed with SFTSV-associated encephalitis/encephalopathy (Fig. 1). Among them, 10 cases performed cerebrospinal fluid (CSF) examination (CSF SFTSV-RNA test was positive in 4 cases, negative in 3 cases, and not available in 3 cases).

Table 1 presents the differences in demographic characteristics, underlying diseases, clinical manifestations, laboratory finding at admission, comorbidity, treatment and outcome parameters between encephalitis/ encephalopathy and non-encephalitis/encephalopathy patients. 45/109 (41.3 %) cases were male and there were more male patients in encephalitis /encephalopathy group than non-encephalitis/encephalopathy group (p = 0.028). The mean age was 61 years in all patients and no differences between the two groups.

**Table 1:** Clinical characteristics of patients with SFTS-associated encephalitis/encephalopathy

	All SFTS patients (N = 109)	Encephalitis/encephalopathy patients (N = 30)	Non-encephalitis/encephalopathy patients (N = 79)	P
*Demographic characteristics*				
Age, years	60.69 ± 12.53	64.07 ± 10.21	59.34 ± 13.16	0.086
Sex, male (%)	45 (41.3)	18 (60)	27(34.2)	0.028
Days prior to admission (days)	6.95 ± 2.42	6.73 ± 2.30	7.04 ± 2.57	0.559
*Underlying diseases, n (%)*				
Hypertension	24 (22.2)	9 (30)	15 (19)	0.163
Diabetes	12 (11)	6 (20)	6 (7.6)	0.071
*Clinical manifestation, n (%)*				
Fever	109 (100)	30 (100)	79 (100)	–
Headache	44(40.4)	30 (100)	14 (17.7)	< 0.001
Cough	36 (33.3)	15 (50)	21 (26.6)	0.014
Wheezing	33 (30.3)	16 (53.3)	17 (21.5)	0.002
Dyspnoea	10 (9.23)	6 (20)	4 (5.1)	0.013
Vomiting/diarrhoea	10 (9.2)	1 (0.9)	9 (8.3)	0.002
*Laboratory findings at admission*				
Leukocytes (*109/L) (normal range, 4–10)	2.85 (1.70–4.63)	2.80 (1.78–4.55)	3 (1.63–4.68)	0.763
Percentage of neutrophils (%) (normal range, 50–70)	55.10 (39.58–72.60)	58.8 (48.9-74.78)	52.15 (38.03–72.60)	0.167
Percentage of lymphocytes (%) (normal range, 20–40)	34.05 (20.70-45.35)	30.1 (21.35–41.83)	35.8 (20.7-47.23)	0.309
Haemoglobin (g/L) (normal range, 110–160)	127.50 (115–142)	130.5 (118–143)	126.5(114.75-141.25)	0.433
Platelets (*10^9^/L) (normal range, 100–300)	47.50 (32–66.25)	42.5 (29.5-64.25)	49 (33.25–69.75)	0.208
PCT (µg/L) (normal range,0-0.5)	0.30 (10.12–0.86)	0.58 (0.24–3.17)	0.22 (0.1–0.73)	0.005
CRP (mg/L) (normal range,0–8)	6.8 (3.15–15.2)	11.55 (4.15–38.75)	5.4 (3–12)	0.016
ALT (U/L) (normal range, 0–40)	73.2 (51.05–137.2)	83 (60.58-168.43)	71.1 (148.8-120.7)	0.167
AST (U/L) (normal range, 0–40)	159.05 (89.55–264.40)	231.90 (129.93-547.73)	122.3 (88.28–234.75)	0.008
LDH(U/L) (normal range, 109–245)	1679 (1326–2595)	1683 (1245–2788)	1430 (1125–2846)	0.623
CK(U/L) (normal range, 25–200)	1027 (506–1528)	1110 (392–2020)	993 (465–1720)	0.912
Amylase (U/L) (normal range, 25–115)	119 (61–195)	185 (87–266.5)	105 (47–163)	0.003
*Comorbidity, n (%)*				
Respiratory failure	10 (9.2)	6 (20)	4 (5.1)	0.013
Vasopressors use	27 (24.8)	13 (43.3)	14 (17.7)	0.001
Acute kidney injury	17 (15.6)	8 (26.7)	9 (11.4)	0.074
Bacteraemia	12 (11)	8 (26.7)	4 (5.1)	< 0.001
Probable or proven-IPA	38 (34.9)	17 (56.7)	21 (26.6)	0.004
*Treatment, n (%)*				
Immunoglobulin	35 (32.1)	14 (46.7)	21 (26.6)	< 0.001
Ribavirin	102 (93.6)	30 (100)	72 (91.1)	0.097
Methylprednisolone	22 (20.2)	10 (33.3)	12 (15.2)	< 0.001
*Outcome*				
ICU transfer, n (%)	16 (14.7)	11 (36.7)	5 (6.3)	< 0.001
Length of hospital stay (days)	9 (6.5–13)	9 (6–11.25)	10 (7–13)	0.272
In-hospital mortality, n (%)	23 (21.1)	13 (43.3)	10 (12.7)	< 0.001

*PCT* procalcitonin, *CRP* C-reactive protein, *AST* aspartate aminotransferase, *ALT* alanine aminotransferase, *LDH* lactic dehydrogenase, *CK* creatine kinase, *IPA* invasive pulmonary aspergillosis

The most common underlying diseases in SFTS patients were hypertension (22.2 %) and diabetes (11 %), and no differences between the two groups. All patients presented with a high fever (100 %), other common symptoms included headache (40.4 %), cough (33.3 %), wheeze (30.3 %), dyspnea (9.23 %), and vomiting/diarrhoea (9.2 %). Among them, patients with cough, wheezing, dyspnea and vomiting/diarrhoea in encephalitis/ encephalopathy group were significantly higher than those in non- encephalitis/ encephalopathy group (p all < 0.05).

Laboratory findings at admission were also showed in Table 1. The leukocyte count with median 2.85 *10^9^/L decreased significantly but the ratio of neutrophils and lymphocytes remained within the normal range. All cases had decreased platelet count with median 34.05 *10^9^/L. C-reactive protein (CRP) and procalcitonin (PCT) in encephalitis/encephalopathy group were both slightly increased, which was statistically different from that in the non- encephalitis/encephalopathy group (p < 0.05). The median value of serum aspartate aminotransferase (AST) and aspartate aminotransferase (ALT) levels were elevated almost in all cases and AST in encephalitis/encephalopathy group obviously raised than non-encephalitis/encephalopathy group (p = 0.008). The serum amylase level with median 119 U/L was increase remarkably in SFTS patients and higher in encephalitis/encephalopathy group than non-encephalitis/encephalopathy group (p = 0.003).

Comorbidity include respiratory failure (10/109, 9.2 %), vasopressors use (27/109, 24.8 %), acute kidney injury (17/109, 15.6 %), bacteremia (12/109, 11 %), and probable or proven-invasive pulmonary aspergillosis (IPA) (38/109, 34.9 %). The rate of respiratory failure, vasopressors use, bacteremia and IPA in encephalitis/encephalopathy group was significantly higher than that in non-encephalitis/encephalopathy group (p all < 0.05).

Immunoglobulin and methylprednisolone were used in 35/109 (32.1 %) and 22/109 (20.2 %) cases, respectively, and the rate of encephalitis/ encephalopathy group was also significantly higher than those in non-encephalitis/encephalopathy group (p < 0.001). 102 (93.6 %) patients were treated with ribavirin as antiviral treatment in in-hospital period and had no difference between the two groups.

16 (14.7 %) cases were transferred to intensive care unit (ICU) and encephalitis/encephalopathy group had higher rate of ICU transfer (p < 0.001), but the length of hospital stay was no differences in two groups. In-hospital mortality totally was 21.1 % and higher in encephalitis/encephalopathy patients (43.3 %) than non-encephalitis/encephalopathy group (12.7 %, p < 0.001).

**Fig. 2:**
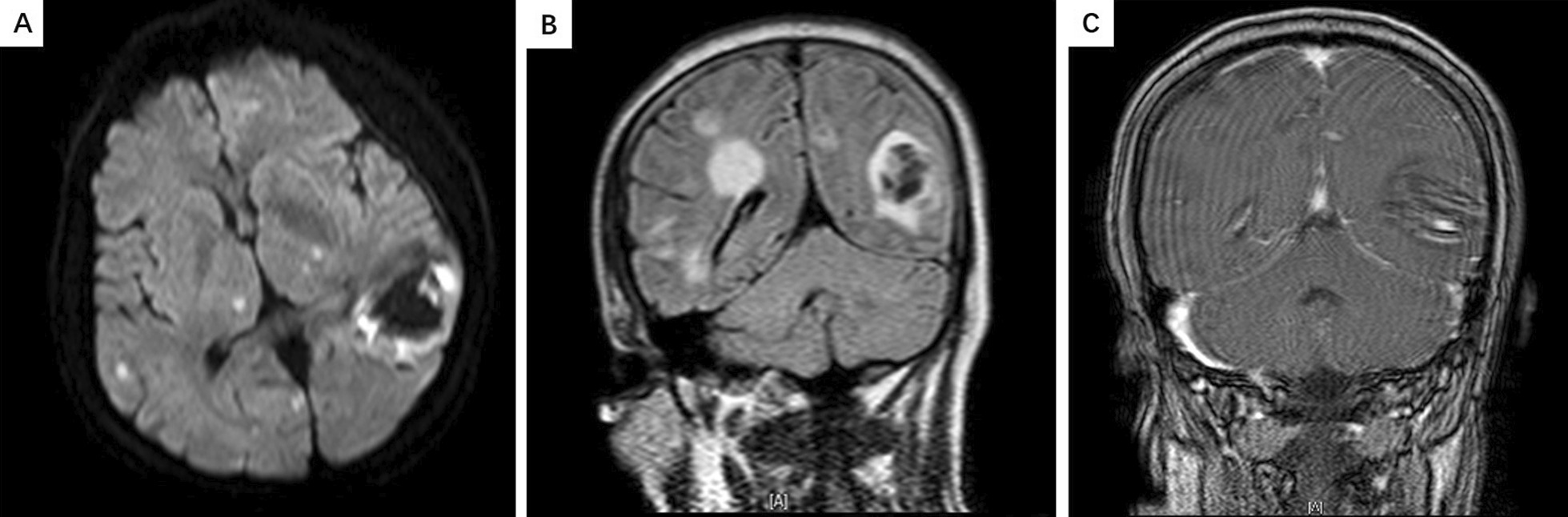
**A** Brain MRI showed patchy mixed signal shadows in the left occipital lobe, with a diameter of approximately 3.9 cm. **B, C** Short T1 signal shadows were visible, and patchy enhancement was observed. Multiple patchy slightly longer T2 signal shadows can be seen in both cerebral hemispheres. DWI phase shows high signal intensity, surrounding areas of oedema, and enhanced patchy enhancement

## Clinical data of SFTS-associated encephalitis/encephalopathy with cerebrospinal fluid (CSF) testing

As shown in Table 2 and 10 cases of confirmed SFTS-associated encephalitis/encephalopathy had performed CSF testing. 2 (20 %) cases were elevated in count of white blood cell (WBC) and 7 cases (70 %) of neutrophil percentage were raised in CSF testing. The protein level in CSF of 9 cases went up dramatically and case 9 is within normal range. 4 cases (40 %) were elevated and 2 cases (20 %) were decreased in glucose level of CSF. There were no obvious abnormalities in CSF chlorine levels in 10 cases. Elevated IgG level of 2 cases was observed. Among the 10 cases, CSF-SFTSV-RNA load of 7 cases were available and 4 cases were positive and 3 cases negative. Compared to blood-SFTSV-RNA load, CSF-SFTSV-RNA load of the 4 positive cases were all obviously lower. Of 10 cases, brain imaging of 9 cases was available and all were abnormal. Among them, the most common brain CT findings were lacunar infarction (6/9), and the most brain MRI findings were hypoxic changes in white matter (3/4). In case 7, intracerebral hemorrhage (ICH) was shown on brain CT scan, and multiple abnormal signals were shown on brain MRI scan, simultaneously. Combined with confirmed IPA diagnosis in this patient, the possibility of intracranial fungal infection was considered (Fig. 2). Of the 10 cases, 8 were co-infected, 7 were IPA, 5 were bacteremia, 4 were combined with IPA and bacteremia. Nine of the 10 cases died (Table 2).

**Table 2:** Clinical presentation, CSF findings, SFTS-DNA testing and imaging findings in patients with SFTS-associated encephalopathy/encephalitis who underwent CSF testing (n = 7)

Case no.	Age /sex	Days to neurological presentation	Neurological presentation	CSF findings								Blood SFTSV- RNA load (copies/ml)	Brain CT	Brain MRI	Co-infection	Outcome
				WBC (*10^6^/L)	Neutrophils (%)	Lymphocyte (%)	Protein (mg/L)	Glucose (mmol/L)	Chlorine (mmol/L)	IgG (mg/L)	SFTSV-RNA load (copies/ml)					
			Normal range	0–8	0–6	40–80	140–450	2.5–4.5	120–132	4.8–58.6	Negative	Negative				
1	61/M	6th day	Unconsciousness, seizure	9.0	35	65	736	5.14	130.9	187	10^3^/L	10^6^/L	Lacunar infarction	Chronic hypoxia changes in white matter	IPA	Died
2	71/M	4th day	Unconsciousness	6.0	66.6	33.4	976	2.41	123	148	10^3^/L	10^4^/L	Lacunar infarction	Chronic hypoxia changes in white matter	IPA, bacteremia	Died
3	71/F	4th day	Altered mental status, disorientation	5.0	20	80	469	3.22	130	NA	10^3^/L	10^6^/L	Lacunar infarction	NA	IPA	Died
4	64/M	4th day	Unconsciousness, seizure	1.0	0	100	572	3.97	126.2	53.1	10^3^/L	10^6^/L	Lacunar infarction	NA	Bacteremia	Died
5	45/M	7th day	Headache, altered mental status	1.0	0	100	542	3.26	133	29.5	Negative	10^6^/L	Lacunar infarction	Chronic hypoxia changes in white matter	No	Survived
6	76/F	3rd day	Unconsciousness	0	0	0	914	5.35	135.3	61	Negative	10^8^/L	NA	NA	No	Died
7	28/M	5th day	Headache, altered mental status	50	36	54	1226	1.33	129	NA	Negative	10^8^/L	ICH	Multiple abnormal signal shadows	IPA	Died
8	61/F	4th day	Headache, altered mental status	3	33.3	66.7	498	9.77	119	NA	NA	10^6^/L	Normal	NA	IPA, bacteremia	Died
9	49/M	6th day	Headache, altered mental status	4	25	75	416	4.02	126	NA	NA	10^6^/L	NA	NA	IPA, bacteremia	Died
10	75/M	4th day	Headache, altered mental status	7	50	50	904	6.16	138	NA	NA	10^4^/L	Lacunar infarction	NA	IPA, bacteremia	Died

*SFTS* severe fever with thrombocytopenia syndrome, *CSF* cerebrospinal fluid, *WBC* white blood cell, *ICH* intracranial haemorrhage, *NA* not available

### Univariate and multivariate analyses of risk factors for the development of encephalitis/encephalopathy for SFTS patients

Univariate logistic regression showed that cough, wheezing, dyspnoea, respiratory failure, vasopressors use, bacteremia, IPA diagnoses, PCT > 0.5 ug/L, CRP > 8 mg/L, AST > 200 U/L and serum amylase level > 80 U/L were the risk factors for the development of encephalitis/encephalopathy for SFTS patients. Multivariate logistic regression analysis identified bacteremia, PCT > 0.5 ug/L and serum amylase level > 80 U/L as independent predictors of encephalitis/encephalopathy development for SFTS patients (Table 3).

**Table 3:** Univariate and multivariate logistic regression analyses of risk factors for the development of encephalitis/encephalopathy in patients with SFTS

	Univariate		Multivariate	
	OR (95 % CI)	*P* value	OR (95 % CI)	*P* value
Sex, male	2.091 (0.888–4.923)	0.091		
Hypertension	1.829 (0.699–4.786)	0.219		
Diabetes	3.042 (0.896 ~ 10.323)	0.074		
Cough	2.762 (1.154 ~ 6.609)	0.022		
Wheezing	4.168 (1.702 ~ 10.209)	0.002		
Dyspnoea	3.700 (1036–13.215)	0.044		
Vomiting/diarrhoea	3.729 (0.452 ~ 30.780)	0.222		
Respiratory failure	5.707 (1.533 ~ 21.240)	0.009		
Vasopressor use	5.306 (2.113 ~ 13.326)	< 0.001		
Bacteraemia	9.375 (2.659 ~ 33.053)	< 0.001	7.211 (1.459–35.635)	0.015
Probable or proven-IPA	3.612 (1.501 ~ 8.689)	0.004		
PCT > 0.5 µg/L	2.581 (1.072 ~ 6.213)	0.034	4.008 (1.110–14.479)	0.034
CRP > 8 mg/L	3.437 (1.435 ~ 8.236)	0.006		
AST > 200 U/L	4.771 (1.950 ~ 11.672)	0.001		
Amylase > 80 U/L	7.929 (2.222 ~ 28.290)	0.001	9.094 (1.761–46.935)	0.008

*PCT* procalcitonin, *CRP* C-reactive protein, *AST* aspartate aminotransferase

## Discussion

This retrospective study showed that 27.5 % of SFTS patients admitted to our hospital developed encephalitis/encephalopathy in our hospital from January 2016 to July 2020, while 13–34 % in other studies [10, 11, 14]. The fatal outcome of the encephalitis/encephalopathy patients was high as 43.3 % in our study while 44.7 % reported in others [10].

There are few data on the CSF data of patients with SFTS-associated encephalitis/encephalopathy. Cui et al. reported that 103 (19 %) of 538 SFTS patients developed encephalitis, and evidence of SFTSV was found in CSF in two patients [10]. The protein and glucose levels in the CSF of the two patients with positive-SFTSV were slightly increased. Park et al. also reported that 14 (34 %) of 41 SFTS patients developed encephalitis but revealed normal protein and glucose levels but plecytosis in all the six patients with positive-SFTSV [11]. The copy number of SFTS virus in CSF in the six cases was lower than that in serum. However, Kim et al. found that on the 12th day of the course of disease, the copy number of virus in CSF was higher than that in serum, indicating that SFTSV was neurotropic [15]. In our study, among the 4 patients with positive-SFTSV in CSF, only one patient had plecytosis, but all the 4 patients had mildly elevated protein level and 2 patients with elevated IgG in CSF. The SFTSV-RNA viral load in CSF was also lower than that in serum. These results indicate that SFTSV direct infection is one of the important mechanisms of SFTSV-associated encephalitis/encephalopathy.

Some studies showed that SFTS patients occasionally suffered from multiple organ failure, with abnormal serum levels of various pro-inflammatory cytokines, which were related to disease severity and mortality in the acute stage of infection [16, 17]. In Nakamura et al. study, the levels of interleukin-10 (IL-10), interferon-gamma (IFN-γ) and interferon induced protein 10 (IP-10) were significantly increased in patients with acute stage of SFTV infection [18]. In other studies, hemophagocytes were found in CSF of patients with SFTSV-associated encephalitis, suggesting that cytokine and chemokine storms may indirectly lead to the disturbance of consciousness in SFTS patients [19, 20]. CNS involvement is closely related to high cytokine and viral load in serum. The high cytokine level caused by viral infection leads to increased vascular permeability, and SFTSV can enter the nervous system through the blood-brain barrier and cause intracranial infection, which may be an important cause of SFTS-associated encephalitis/encephalopathy.

SFTS-associated encephalitis/encephalopathy is also related to the immune state of patients. In some studies SFTSV was not detected in CSF or even in some autopsy cases [12, 19, 21, 22]. CD4 + T cells decreased significantly in blood of patients in the acute stage of SFTSV infection, while CD8 + T cells increased or did not change significantly, accompanied by dramatic decrease in NK cells, while the number of CD4 + and CD8 + T cells further decreased in blood, while the number of NK cells further increased. When the cellular immune function is impaired, mainly mediated by CD4 + and CD8 + T cells, the immune transition response caused by the high activation of NK cells is an important pathological basis for multiple organ injury including CNS in severe SFTS patients. Furthermore, the increase of B cells in blood of severe SFTS patients is more obvious, and B cells can further differentiate into plasma cells [23]. The recent study found that within 3 weeks after the onset of SFTS, the proportion of plasma cells in non-survivals to B cells was higher than survivors, but they were non-functional plasma cells [24]. Apoptosis of monocytes in the early stage of SFTSV infection in the non-survivors reduced the antigen presentation of DC and affected the differentiation and function of CD4 + T cells, which was an important cause of virus-specific humoral immunodeficiency in non-survivors.

In our study, multivariate analysis identified 3 variables (bacteremia, PCT > 0.5ug/L and serum amylase level > 80U/L levels) as independent predictors for the development of encephalitis/encephalopathy in patients with SFTS. However, no other published manuscripts have found that these three factors associated with the incidence of SFTS-associated encephalitis/encephalopathy, but according to the pathogenesis of SFTS-associated encephalitis/encephalopathy, cytokine and chemokine storms lead to the increase of capillary permeability, secondary infection such as gut-origin sepsis, so bacteremia is independent risk factors for the development of SFTS-associated encephalitis/encephalopathy. The mechanism of hyperamylasemia in SFTS-associated encephalitis/encephalopathy patients is not clear. Studies have shown that cytokine storm played a key role in the development of the occurrence of acute pancreatitis, main show is hyperamylasemia, therefore, may be higher serum amylase level may be associated with cytokine storm [25, 26], Other studies have also found that the application of ribavirin may also lead to hyperamylasemia as a side effect [27, 28]. Therefore, to a certain extent, these three independent risk factors (bacteremia, PCT > 0.5 ug/L and serum amylase level > 80U/L levels) for the development of SFTS-associated encephalitis/encephalopathy reflect the severity of cytokine storm. The more severe the cytokine storm, the greater the chance of SFTS-associated encephalitis/encephalopathy. These three variables were routinely evaluated in clinical practice, thus yielding a highly predictive value for discriminating the patients at higher risk of encephalitis/encephalopathy, who should get more attention in treatment.

The specific treatment of SFTS is still unknown. In 2012, the Ministry of Health of China recommended ribavirin intravenous therapy (500 mg per day) in SFTS Treatment Guidelines. Ribavirin is a synthetic nucleoside broad-spectrum antiviral drug that has inhibitory activity against both DNA and RNA viruses [29]. In recent years, more and more clinical data have shown that ribavirin does not increase platelet count nor reduce serum viral load [8, 14, 30]. The in vitro experimental study conducted by Shimojima et al. compared the antiviral efficacy of ribavirin before and after 3 days of SFTSV inoculation, and found that ribavirin used before inoculation could significantly inhibit the proliferation of the virus, while the inhibitory ability of antiviral drugs decreased significantly after inoculation. Therefore, it is speculated that ribavirin is more suitable for the post-exposure prophylaxis of SFTS [31]. Because the virus into the blood is still in the incubation period, the patient has no clinical symptoms, no treatment measures. After 5–14 days of incubation and onset, the virus has already multiplied in large numbers in cells, and ribavirin can no longer reverse virus replication. It is inferred that the poor treatment effect of ribavirin may be due to missing the optimal treatment time.

Recently, studies have conducted some new treatment attempts for SFTS patients. More attempt to treat SFTS-associated encephalitis/ encephalopathy patients is corticosteroid pulse therapy. Corticosteroid pulse therapy can inhibit the overproduction of cytokines, thus reducing organ failure [32]. However, it is worth noting that steroid therapy may not always be beneficial for secondary infection complications (such as IPA) of SFTS patients [33]. However, the number of lymphocytes in the early stage is significantly reduced in SFTS patients [34], and the use of steroid can lead to further reduction of lymphocytes, which is not benefit to the body’s immune function. Gamma-globulin has been shown to effectively inhibit macrophage activation and cytokine storms in Crimean Congo haemorrhagic fever virus infection [35]. The application of gamma globulin in the treatment of severe SFTS patients has been reported [36]. The mechanism is to block virus replication by complementing non-specific anti-virus, anti-bacterial and anti-other pathogens IgG antibody in the body, thus playing a role of neutralizing toxin. Further evidence on the application of gamma globulin in the treatment of SFTS patients needs to be accumulated. Another attempt of plasmapheresis for SFTS patients has been reported recently [18, 37–39]. Studies have shown that plasmapheresis can reestablish homeostasis and improve the coagulation status of patients. To date, steroids are mostly used in combination with plasma exchange, immunoglobulin and antiviral drugs, which makes it difficult to distinguish the effects of these therapies for SFTS patients.

Our study has several limitations. First, there were a relatively small number of cases because of the relatively low incidence of SFTS-associated encephalitis/encephalopathy. Second, the study was a retrospective study, CSF-SFTSV-RNA screening tests were not available for all patients. Finally, the study is a single-center study and the results may be limited.

## Conclusions

This study showed that there was a high incidence and mortality of encephalitis/encephalopathy in SFTS patients, it is necessary to strengthen the treatment of SFTS patients with neurological symptoms. SFTS patients with bacteremia, PCT > 0.5 ug/L or serum amylase level > 80U/L should be warned to progress to encephalitis/encephalopathy.

## Data Availability

The datasets generated and/or analyzed during the current study are not publicly available due individual privacy of patients could be compromised, but are available from the corresponding author on reasonable request.
